# Can the Outputs of LGN Y-Cells Support Emotion Recognition? A Computational Study

**DOI:** 10.1155/2015/695921

**Published:** 2015-06-15

**Authors:** Andrea De Cesarei, Maurizio Codispoti

**Affiliations:** Department of Psychology, University of Bologna, Viale Berti Pichat 5, 40128 Bologna, Italy

## Abstract

It has been suggested that emotional visual input is processed along both a slower cortical pathway and a faster subcortical pathway which comprises the lateral geniculate nucleus (LGN), the superior colliculus, the pulvinar, and finally the amygdala. However, anatomical as well as functional evidence concerning the subcortical route is lacking. Here, we adopt a computational approach in order to investigate whether the visual representation that is achieved in the LGN may support emotion recognition and emotional response along the subcortical route. In four experiments, we show that the outputs of LGN Y-cells support neither facial expression categorization nor the same/different expression matching by an artificial classificator. However, the same classificator is able to perform at an above chance level in a statistics-based categorization of scenes containing animals and scenes containing people and of light and dark patterns. It is concluded that the visual representation achieved in the LGN is insufficient to allow for the recognition of emotional facial expression.

## 1. Introduction

An influential model of emotion processing suggests that visual information is processed along two pathways: a slower cortical pathway and a faster subcortical pathway [1–7]. Both pathways carry visual information to the amygdala, a subcortical structure involved in emotional processing [1–10]. However, while the cortical pathway to the amygdala has been extensively documented (e.g., [11, 12]), greater debate surrounds the subcortical pathway. It has been suggested that the subcortical pathway connects the lateral geniculate nucleus (LGN) to the superior colliculus, the pulvinar, and finally the amygdala [1, 2], which is ultimately responsible for the detection of emotional stimuli such as fearful faces [5–7]. Importantly, this pathway could allow for emotional recognition under critical visual conditions such as brief exposure, peripheral viewing, low visibility, or cortical lesions.

The available anatomical support for the existence of the subcortical pathway relies on animal studies, which have suggested that in rats a subcortical pathway may mediate fear conditioning to flashes of light or acoustic noise [1–3]. In humans direct evidence of the subcortical pathway is still lacking, but indirect evidence comes from a study on binocular rivalry [13] and from a blindsight patient (patient G.Y. [14]; but see [15–17] for evidence of extrastriate activity in the same patient).

Functional evidence for the existence of a subcortical pathway devoted to emotional processing comes from several studies examining the response to low-passed blurred faces. This evidence stems from the observation that low spatial frequencies can be quickly processed by the fast magnocellular pathway [5, 18–20]. In a highly cited study [5], participants viewed high-passed (HSF) or low-passed (LSF) versions of fearful and neutral faces. The results indicated that LSF fearful faces enhanced the activation of the amygdala as compared to neutral expressions, while HSF faces modulated fusiform activity, irrespective of emotional expression. The authors interpreted these data as supporting a dual route model, in which LSFs travel from the LGN to the amygdala subcortically, while HSFs are analyzed cortically [5]. However, the possibility that LSFs are analyzed cortically is difficult to rule out in intact participants [21], and the high/low spatial frequency distinction in the LGN is complex to achieve experimentally [22].

Taken together, the available anatomical and functional evidence does not allow us to ultimately conclude whether a subcortical pathway devoted to emotional processing exists in humans [21]. Moreover, provided that such a pathway exists, it is necessary to demonstrate that it can carry out the processing necessary to convert the retinal input into stable and invariant representations [23]. To date, there are no data demonstrating that the visual representation that is achieved in the LGN can support visual invariances. In fact, the available evidence concerning the subcortical pathway in rats was demonstrated using stimuli which do not require invariance to be detected, that is, flashes of light and noise bursts [1–3]. Therefore, it is legitimate to doubt that the complex visual processing that is necessary to recognize an emotional expression can be carried out without the involvement of the visual cortex [21, 23–25].

Here we examined the functional properties of the putative subcortical route by means of a computational approach. Using a computational model to estimate the outputs of LGN cells, we investigated whether the visual representation that is achieved can support emotional recognition. When the visual input reaches the LGN, it has not been segmented and converted into an invariant representation by cortical processing in the visual areas. However it is possible that perceptual decisions can still be made based on coarse visual regularities that allow one to distinguish between different contents. Several studies have indicated that some classes of stimuli (e.g., pictures of naturalistic rather than man-made objects) are characterized by visual properties, for instance, in terms of their representation in the Fourier frequency space or in the Weibull distribution of contrasts [26, 27]. These perceptual regularities may be exploited by the visual system to aid categorization. For instance, a relationship between scene statistics (e.g., visual clutter) and content (e.g., presence of an animal) may be due to the fact that animals are likely to appear in visually cluttered environments (e.g., woods). From the perspective of the visual system, detecting a visual regularity that is predictive of the presence of a specific content may constitute an advantage in the detection of that content. However, it must be noted that this statistics-based categorization does not equal semantic categorization, which requires further processing to be achieved [28].

Several studies have investigated how visual information is represented in the LGN [29–32]. The LGN is a subcortical structure which is organized into magnocellular and parvocellular layers. Magnocellular layers convey a relatively more coarse representation of the visual input and contain Y-type cells. Parvocellular layers have a temporally slower but visually more fine-grained representation of the visual input and contain mainly X-type cells. Both the magnocellular and parvocellular layers project to the visual cortex, as well as to other subcortical structures such as the superior colliculus.

A computational model of visual representation in the X- and Y-cells of the LGN has recently been suggested [27], based on the known properties of the visual input representation in the LGN [33–35]. This model computes the beta and gamma parameters of the Weibull fit to the contrast distribution, and these parameters have been shown to correlate with the outputs of the X-cells and Y-cells of the LGN [27]. Based on the distribution of contrast using different spatial scales, this model has been shown to predict cortical activation in the primary visual cortex, which is the first cortical region to be reached by LGN outputs [27]. More specifically, pictures of natural scenes were presented to participants, and event-related electrocortical changes were recorded and CSD-corrected to eliminate the effects of distal sources. A factor analysis was carried out on these data in order to investigate the factors which modulated electrocortical activity on electrode Iz, which overlies the primary visual cortex. The results of these analyses indicated that the gamma parameter explained most of the variance of the event-related electrocortical activity [27]. These data were interpreted as supporting the possibility that the output of LGN cells may modulate the activity in the immediately successive cortical processing stage [27, 36–38]. However, direct evidence is lacking as to whether subcortical structures can recognize emotional expressions based on the outputs of LGN Y-cells.

*The Research Problem*. If the outputs of the LGN Y-cells serve as an input to other subcortical structures that are devoted to emotion recognition, then it can be argued that the representation of visual information at this stage is sufficient to discriminate emotional from neutral facial expressions. Is this really so? Here, we tested this possibility by training an artificial classificator with inputs from Y-cells of images of fearful or neutral facial expressions. In a successive testing phase, it was ascertained whether the classificator could learn to discriminate fearful from neutral faces [39]. Moreover, we assessed whether a low noise with 1/*f* amplitude distribution could interfere with emotional facial expression recognition.

Additionally, it has been suggested that emotion recognition in the subcortical route is supported by low spatial frequencies and that fuzzy stimuli containing low, but not high, spatial frequencies are processed via this route [5, 18–20]. Here, we addressed this possibility by testing the classificator with pictures of faces that could either be intact or composed of low spatial frequencies (LSFs) or high spatial frequencies (HSFs). Straightforward predictions may be put forward concerning the conditions under which emotional expressions should be discriminated. First, emotional expressions should be discriminated in the intact and LSF conditions. This is supported by a number of studies reporting that intact as well as LSF faces modulate electrocortical activity or performance in attentional tasks [5, 18–20]. Similarly, no emotional recognition for HSF faces should be expected, as it has been suggested that emotion recognition in the subcortical route is supported by the magnocellular system, which is almost blind to high spatial frequencies.

The present study is organized into four experiments. In Experiment 1 we tested whether facial expressions could be sorted into fearful and neutral ones. In Experiment 2 we asked whether pairs of faces showing the same or a different expression could be sorted into matching and mismatching pairs. In Experiment 3, we examined whether Y-cell outputs alone are sufficient to sort natural scenes into those representing animals or people, as was previously observed for the combined output of X-and Y-cells [27, 36, 37, 40]. Finally, based on the observation that the subcortical route should be sensitive to abrupt light changes [1, 2], in Experiment 4 we investigated whether the computational model was able to discriminate light from dark patterns.

## 2. Methods

### 2.1. Stimuli (Experiments 1 and 2)

A total of 140 pictures of faces were selected from the Karolinska Directed Emotional Faces database (KDEF [41]). These faces portray an equal number of male and female faces, displaying either a fearful or a neutral expression. Pictures were converted to grayscale and rescaled to 188 (horizontal) × 250 (vertical) pixels, corresponding to 7.52 × 10° of visual angle in the model of LGN that we used. Pictures were shown in the intact version, masked by 1/*f* visual noise, and filtered with a low-pass filter (LSF) or filtered through a high-pass filter (HSF).

In the intact condition, grayscale stimuli were presented without any filter or noise. In the noise condition, a visual noise with 1/*f* amplitude distribution was generated and added to the image, according to a noise/image ratio of 1/5 (one part of noise per four parts of image).

For the LSF and HSF versions, the spatial frequency filters were as follows. The low-pass filter passed all spatial frequencies up to a threshold value F0, then declined parabolically with log spatial frequency, reaching zero at spatial frequency F1 = 3·F0, and prevented all frequencies higher than F0 from being displayed [42–44]. The high-pass filter acted in a symmetrical way, allowing all frequencies higher than a threshold value to pass and stopping all frequencies three times lower than the threshold value. Here, the LSF used a F0 threshold of 5.33 cycles per image (cpi), while the HSF had a threshold of 24 cpi.

### 2.2. Estimation of Responses of LGN Y-Cells

Based on the literature supporting the subcortical route to the amygdala, we were interested in examining the output of Y-cells, as these are present in the magnocellular layer of the LGN. In previous studies and computational work, it was observed that the gamma parameter of the Weibull fit to the distribution of contrast at a relatively coarse scale approximates the output of Y-cells in the LGN well, while the beta parameter at a finer scale approximates the output of X-cells [24]. Therefore, we estimated the output of Y-cells in the LGN using an algorithm based on the Weibull fit to the distribution of contrasts [36].

A set of filters spanning 5 octaves was used (standard deviation, in pixel = 5, 10, 20, 40, and 80). The output of each filter was normalized with a Naka-Rushton function with 5 semisaturation constants between 0.15 and 1.6 to cover the spectrum from linear to nonlinear contrast gain control in LGN. Then, one filter response was selected for each location in the image using minimum reliable scale selection [36, 45]. The thus-obtained contrast magnitude map was converted into a 256-bin histogram summarizing the contrast distribution of the image, and the Weibull fit to this distribution was calculated using a maximum likelihood estimator (MLE). Finally, the gamma parameter of the Weibull fit represents the shape of the distribution of contrasts and was calculated and used for further analysis.

### 2.3. Artificial Classification

**Figure 1:**
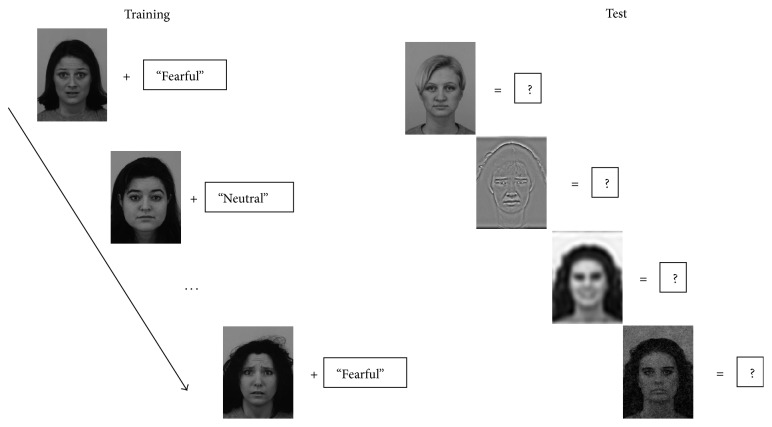
Schematic representation of the training and test procedure for Experiment 1. In the training phase, intact photos of faces showing emotional and neutral expressions are given as input to the support vector machine alongside a label describing their expression. In the test phase, intact and degraded versions of faces are given as input to the SVM, and a decision is required regarding whether the face shows a fearful or a neutral expression.

We trained and tested a support vector machine (SVM) to classify facial expressions, based on the gamma parameter of the Weibull fit to the contrast distribution in the LGN Y-cells. A support vector machine is a classification algorithm dedicated to finding the best separation (hyperplane) between two classes of data, based on one or more continuous predictor variables, and has been used in previous research on visual categorization [39]. The organization of the training and test phase is reported in Figure 1. The algorithm requires an initial training phase, in which the relationship between predictor variables and categorical membership is learned and a model is generated. A test phase then follows in which predictor variables for new data are given as input to the SVM, and the SVM classifies each input in the two output categories. The correspondence between the correct categorical membership and the SVM output labels reflects the degree to which the SVM is able to detect and learn an association between the input variables and the categorical membership. The SVM analysis was performed using MATLAB.

*Experiment 1 (expression recognition task).* In the expression recognition task, we investigated whether facial expressions can be classified as neutral or fearful, based on gamma values. To this aim, the fearful and neutral faces were half-split into a training subset and a test subset, keeping an equal number of male and female faces in each subset. Then, the gamma parameter of the Weibull contrast fit in the training set, along with expression labels, was given as input to a support vector machine (SVM). Importantly, the gamma parameter for training was calculated on the intact version of the face. 140 faces in total were used for training. The learning was later tested on the remaining half of the faces, in the intact as well as degraded (LSF, HSF, noise) conditions.

The training and tests were repeated 100 times, each time with a different subsample of faces selected. The gamma parameter of each face was given as input to the SVM, which classified it as showing a fearful or a neutral expression. The results of these runs were collapsed to obtain averages and confidence intervals for SVM categorization accuracy.

*Experiment 2 (expression matching task).* In the same/different expression task, we asked whether the gamma parameter could be used to discriminate whether two faces show the same or different expressions. To this aim, we created 128 pairings of faces matched for expressions (both faces showing a neutral or a fearful expression) and pairings in which the expression mismatched (one neutral and one fearful, or vice versa). In all pairings, the gender of both faces was the same. Half of these pairings (*n* = 64) were used for training and the other half for testing. For each pairing, the gamma parameter of the Weibull contrast fit of the two images in the intact condition, along with a label indicating match or mismatch, was given as input to the SVM. The learning was later tested on the remaining half of the faces, in all conditions (intact, noise, LSF, and HSF).

The procedure involving pairings, training, and test was repeated 100 times, each time creating new pairings and selecting different pairings for training and test. During testing, the gamma parameter of face pairings in all conditions was given as input to the SVM, which classified them as matching or mismatching. The results of these runs were collapsed to obtain an overall index of accuracy.

*Experiment 3 (animal/people categorization task).* In the animal/people categorization task, we aimed to test whether our model could discriminate natural scenes depicting humans or animals based on Y-cell outputs only. A total of 280 stimuli (animals = 140, people = 140) were selected from the Internet and modified in the same way that was described for faces. Picture permutation, as well as training and test of the SVM, proceeded in the same way that was described for Experiment 1. Accuracy and confidence intervals of the classification were calculated for each of the 100 repetitions of the procedure.

*Experiment 4 (light/dark categorization task).* In the light/dark categorization task, we investigated whether our model could discriminate light from dark patterns. A total of 280 stimuli (sized 480 × 480 pixels) were generated by creating circles of varying line width (ranged from 2 to 20 pixels), radius (ranged from 20 to 240 pixels), and color (white circle on black background or vice versa). These stimuli were then low-pass filtered using a threshold of 5 cycles per image (cpi). Picture permutation, as well as training and testing of the SVM, was carried out in the same way as in all other experiments. Accuracy and confidence intervals of the classification were calculated for each of the 100 repetitions of the procedure.

### 2.4. Statistical Analysis

For each type of analysis, the results of the 100 runs of the training-test pairing were aggregated to obtain a mean and a confidence interval of the categorization accuracy of the SVM during the test, separately for each condition (intact, noise, LSF, and HSF). Due to the artificially high number of samples the significance of the *p* value is inflated; for this reason, we report confidence intervals, since this measure is not inflated by the high number of repetitions of the procedure [46, 47]. As the present experiments aim to assess whether the SVM can learn to categorize faces and scenes at a level above chance, for each condition we determined whether the 95% confidence interval included the chance level (0.50) or not.

## 3. Results

**Figure 2:**
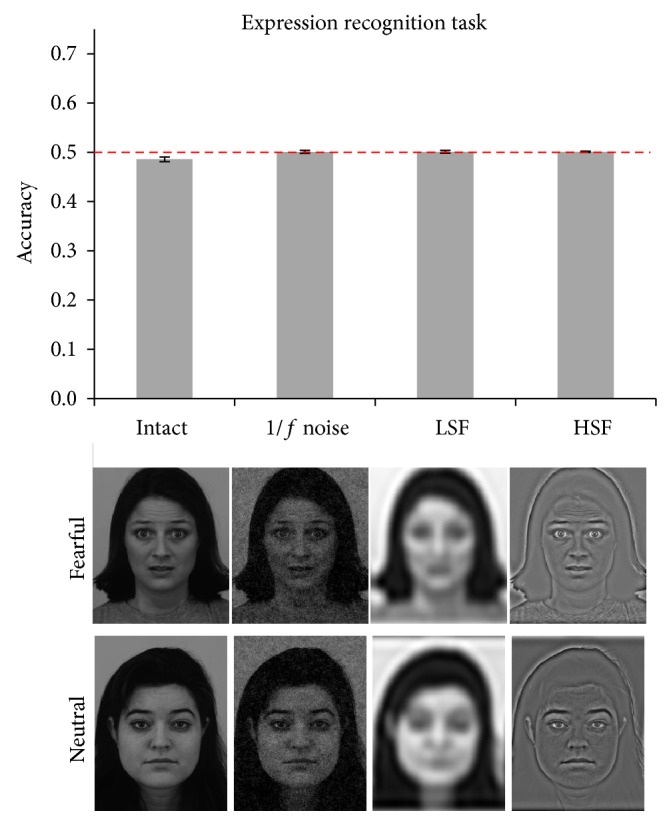
Results of Experiment 1. Bars represent average accuracy in all four conditions, and error bars represent 95% confidence intervals. In this subsequent figures, the dashed red line represents chance level. Below each bar, examples for faces in each of the conditions are reported.

*Experiment 1 (expression recognition task).* In the expression categorization task, we asked whether facial expressions could be sorted into fearful and neutral categories using Y-cell information. Accuracy was at chance in all conditions (Figure 2). Moreover, all conditions yielded accuracy levels within the same confidence intervals (minimum CI = 0.48, maximum CI = 0.51).

The results of Experiment 1 seem to indicate that the output of Y-cells cannot be used to distinguish fearful from neutral faces when they are presented alone. However, one could wonder whether the model may recognize emotional expressions at a level that is above chance if it is given a template of a “fearful” and a “neutral” expression. It has been suggested that such a template exists, as an evolution-shaped circuitry that mediates response and learning to fearful stimuli, including emotional expressions [48]. This possibility requires the current input representation at the LGN stage to contain enough information to match a facial expression to a template. If this is so, then it can be expected, based on the same information, that the system can decide whether two faces show the same expression or not. We examine this possibility in Experiment 2, in which two faces are compared, and it is tested whether their expressions match or mismatch. The rationale of Experiment 2 is that, in a two-stimulus comparison, each facial expression serves as a template for the other one.

**Figure 3:**
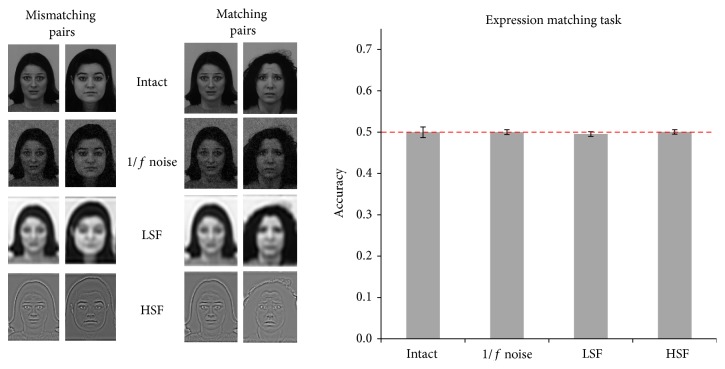
Results of Experiment 2. On the left, examples of matching and mismatching pairs are reported. In the bar plot, accuracy in all four conditions with 95% confidence intervals is reported.

*Experiment 2 (expression matching task).* In Experiment 2, the same/different expression task was carried out by the SVM. Similar to Experiment 1, neither the intact nor any of the degraded conditions differed from chance and no difference between degradation conditions was observed (Figure 3). Mean accuracy was 0.50, with minimum CI = 0.49 and maximum CI = 0.51.

The results of Experiment 2 further support the view that information as represented in Y-cells of the LGN is insufficient to discriminate between fearful and neutral faces, even when a template is provided. However, previous computational studies used both the beta and the gamma parameters to simulate the outputs of X-cells (parvocellular layer) and Y-cells (magnocellular layer) to the visual cortex and observed that these parameters predicted both accurate categorization of natural scenes and ERP amplitude over the primary visual cortex [27]. On the other hand, here we only used the output of Y-cells, and it may be that this information alone is insufficient to discriminate any visual content. For this reason, we conducted a third experiment in which we asked the SVM to classify natural scenes into those representing animals or people, using only Y-cell outputs.

**Figure 4:**
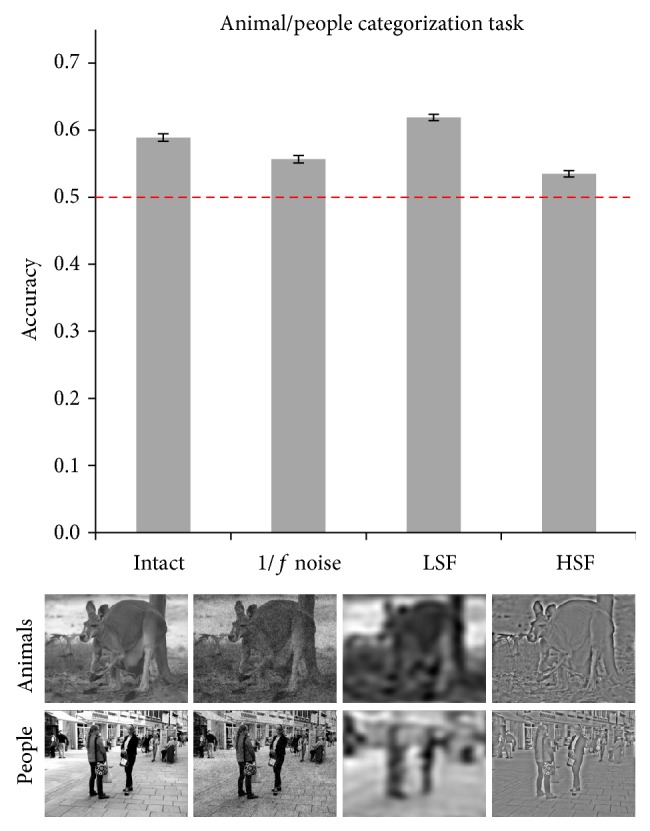
Results of Experiment 3. Accuracy in all four conditions is reported with error bars representing 95% confidence intervals. Below each bar, examples for the animal and people categories are reported.

*Experiment 3 (animal/people categorization task).* In Experiment 3, we asked whether natural scenes could be parsed into those representing animals and people, using only Y-cell outputs. The results are reported in Figure 4 and show that in the intact condition scenes containing animals could be discriminated from scenes containing people at a level above chance (accuracy = 0.59, minimum CI = 0.58, and maximum CI = 0.59). Interestingly, accuracy was above chance in all degraded conditions (noise = 0.56, LSF = 0.62, and HSF = 0.53). Confidence intervals indicate that accuracy decreased when adding 1/*f* noise (minimum CI = 0.55, maximum CI = 0.56) and when scenes were high-pass filtered (minimum CI = 0.53, maximum CI = 0.54) as compared to intact stimuli. On the other hand, low-pass filtering increased accuracy compared to all other conditions (minimum CI = 0.61, maximum CI = 0.62).

The results of Experiment 3 indicate that Y-cell outputs can be used to classify natural scenes as depicting animals or people. Importantly, this classification does not equal semantic categorization but represents a process that is driven by regularities in the perceptual appearance of images. By exploiting these regularities, the visual system can enhance categorization performance. However, in animal studies, which suggested that a subcortical pathway may support fear conditioning, light bursts were used as conditioned stimuli [1, 2]. Therefore, a model of LGN response should be able to detect abrupt changes in illumination. To this end, we designed Experiment 4, in which simple stimuli consisting of white circles on a black background, or black circles on a white background, were presented to the classificator. The model was expected to be able to discriminate light patterns (black circle on white background) from dark ones (white circle on black background).

**Figure 5:**
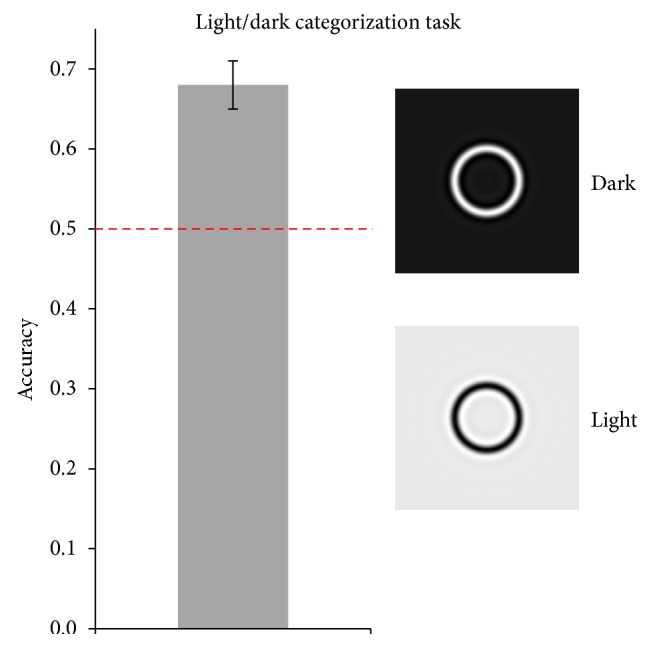
Results of Experiment 4. Accuracy for the light/dark categorization task is reported, along with error bars representing 95% confidence intervals. On the right, examples of the light and dark stimuli are reported.

*Experiment 4 (light/dark categorization task).* In the light/dark categorization task, we presented black and white patterns and asked the model to categorize them as light or dark. It was observed (Figure 5) that the model categorized stimuli above chance level (minimum CI = 0.65, maximum CI = 0.71).

## 4. Discussion

Here we aimed to examine the functional properties of the putative subcortical route with a computational approach, namely, by investigating whether the visual representation of the Y-cells of the LGN may support emotion recognition by an artificial categorization system. The result is a clear negative answer, concerning both the ability of the system to sort faces into fearful and neutral expressions (Experiment 1) and to detect a match between two facial expressions (Experiment 2) and the possibility that some recognition could be achieved under degraded visual conditions such as LSF (Experiments 1 and 2). In contrast, positive findings were observed concerning the categorization of light and dark patterns and the statistics-based categorization of natural scenes (Experiments 3 and 4).

These data are in agreement with previous suggestions that complex computations are necessary for object identification, including recognition of emotional stimuli [21, 23, 49]. As the retinal projection of distal objects is continuously changing, the computing of visual invariances is a necessary step to convert the fleeting retinal projection into a stable perceptual representation. Human and animal research has demonstrated that size and position invariance is first achieved in the anterior inferotemporal cortex [50, 51]. Moreover, it should be noted that the stimuli that were demonstrated to be efficiently processed subcortically in rats during fear learning were acoustic noises and flashes of light and did not require visual invariances to be detected [1–3]. Here, we observed that the simple LGN model tested could categorize light and dark patterns; the ability to discriminate abrupt light changes is consistent with a role of LGN Y-cells in fear learning for simple stimuli such as light bursts [1, 2]. However, the same model could not discriminate emotional expression of faces, neither in a single-category test nor in the same/different expression test. Altogether, these data do not support the view that complex stimuli such as facial expressions may be processed via a purely subcortical route.

The amygdala is highly interconnected with several areas of the cortex, including prefrontal areas and visual associative areas [11, 12]. Therefore, it is possible that the critical information required to recognize and respond to emotional stimuli does not come from a subcortical route, but it is conveyed to the amygdala through one (or more) of many cortical routes. This possibility has recently been put forward by some of the proponents of the subcortical route [52]. Once visual information concerning a stimulus has been cortically processed and reaches the amygdala, it is a hallmark finding that amygdala activity increases for stimuli that are novel, motivationally significant, and either positively or negatively valenced [8–10].

The present data suggest that the coarse visual representation that is achieved in the LGN cannot support the processing of emotional facial expressions. More complex computations are likely to be necessary for the processing of even relatively simple stimuli such as human faces. Natural scenes, such as those representing threats, and appetitive stimuli in real environments are likely to require even more complex stages of processing to be identified and converted into a coherent percept with associated motivational value and therefore cannot benefit from putative subcortical processing based on LGN information [21, 23–25, 49]. Moreover, these data cast doubt on the possibility that at the LGN processing stage a portion of the face (e.g., the eyes) may be selected and parsed to identify emotional expression. More specifically, selecting and parsing a portion of the face should require some kind of visual invariance (e.g., location and size), but invariances are only reached at later processing stages in the inferotemporal cortex [50, 51]. Finally, the behavioral repertoire which is needed to adaptively respond to real emotional scenes is likely to be more complex compared to that expected based on the detection of presence or absence of a relevant stimulus [25]. As visual processing proceeds and the visual input is converted into a semantic representation of the scene, the modulation of cortical and subcortical structures can be observed and adaptive responses may be selected. These data invite caution against the possibility of amygdala activation, which can be elicited by cortical as well as subcortical inputs, being mistaken for subcortical processing [21].

Finally, in the present study, we observed that the output of Y-cells alone can be used to categorize complex scenes into those representing animals or humans. It should be noted that this type of categorization does not equal a semantic categorization of the content of a scene, but rather the detection of a perceptual regularity (e.g., parameters of the Weibull fit) that is associated with the presence of a specific content (e.g., animals). These data are informative in two ways. First, they show that, consistent with previous studies [36–38, 40], biologically significant scene statistics may modulate scene processing in specific categorization tasks, such as deciding whether a scene contains an animal. Second, they indicate that when diagnostic visual information is present, the present model based on Y-cells can use it to categorize visual input. Concerning natural scenes, the ability to categorize acquired during training with intact scenes transferred to all degraded conditions, suggesting that each of these conditions preserves diagnostic information that is usable to discriminate animals from people based on simulated Y-cell outputs. Interestingly, in this respect, we observed here that accuracy was reduced by 1/*f* noise and by high-pass filtering, while it was enhanced by low-pass filtering, compared to when scenes were intact. These data indicate that the low spatial frequencies were most diagnostic for the present scene categorization task and that the presence of 1/*f* noise, as well as of high spatial frequencies, dampened the categorization of natural scenes.

*Limitations and Future Directions.* The present study addressed, using the simulated output of Y-cells of the LGN, whether recognition of emotional expression is possible at that processing stage. While the response was clearly negative, some limitations must be acknowledged.

First and most important, we used a simulation of the visual representation in the LGN. More specifically, we computed the Weibull fit to distribution of contrast using parameters that approximate the output of LGN Y-cells and examined whether the results of this fit can aid emotional expression recognition. To date, it is not possible to directly assess visual representation in the LGN, and the simulation carried out here is based on the properties of LGN cells that have been observed in animal and human research examining the receptive fields of cells at several stages of visual processing [27, 29–35]. It is possible that the simulation does not entirely capture the LGN representation of the visual input, and the simulations which were run here might be repeated once new models of visual representation in LGN are available.

Second, it is possible that the visual representation which is achieved in the LGN is further processed before reaching the amygdala, thus unveiling properties of the visual input that are latent at the stage examined here. However, no such mechanism has been demonstrated to date, and it is a challenge to the proponents of alternative neural models to demonstrate how these models can carry out the necessary computations.
